# Evaluating the Novel Coronavirus infection outbreak surveillance results in a state hospital: a retrospective study

**DOI:** 10.4314/ahs.v21i3.19

**Published:** 2021-09

**Authors:** Ezgi Dirgar, Betül Tosun, Soner Berşe, Nuran Tosun

**Affiliations:** 1 Hasan Kalyoncu University, Faculty of Health Sciences, Nursing Department, Gaziantep, Turkey; 2 Gaziantep University, Faculty of Health Sciences, Nursing Department, Gaziantep, Turkey

**Keywords:** COVID-19, coronavirus, surveillance, retrospective study

## Abstract

**Background:**

Coronavirus disease (COVID-19) has raised the global public health concern and has been declared a pandemic by the World Health Organization.

**Objectives:**

This study was aimed to examine the clinical course and outcomes of the patients with COVID-19 in the southeastern part of Turkey.

**Methods:**

This retrospective study was conducted on the files of 173 patients who were diagnosed with COVID-19. The “COVID-19 Case Information Form” in the patients' medical records was used.

**Results:**

Of the patients with COVID-19, 64.2% were male and 16.2% had a chronic disease. Their mean age was 34.76±25.75 years. Cough and fatigue were the most common clinical symptoms at admission with 38.7%. The patients at the age of 65 and over were treated mostly in the intensive care unit, and the symptoms associated with the cardiovascular and nausea and vomiting were observed more often (p<0.05).

**Conclusions:**

It was found that the majority of the patients were male and there were differences between the age groups in terms of transmission route, the clinic where they were being followed-up, some symptoms, and clinical status outcome. It is recommended that multi-center, prospective, experimental, or observational studies with larger samples should be and the patients should be followed-up for longer periods.

## Introduction

In December 2019, several cases of pneumonia of unknown origin were reported in Wuhan, the capital of Hubei Province, China, and the novel coronavirus identified to cause the pneumonia was named Coronavirus Disease-19 (COVID-19) by World Health Organization (WHO) 1. The disease has spread rapidly to mainland China and then to the world. WHO declared the Public Health Emergency of International Importance (PHEIC) on January 30, 2020, and a pandemic on March 11, 2020^2^.

Centers for Disease Control and Prevention (CDC) (2020) have reported that the common symptoms of the disease are fever, cough, and shortness of breath and, on the other hand, the less common symptoms are weakness, myalgia, sore throat, runny nose, loss of taste and smell, diarrhea and abdominal pain. Moreover, as the number of cases has increased, the observations have shown that the first symptoms are associated not with respiratory distress, but with diarrhea, anorexia, and vomiting 3–5. Computed Tomography (CT) has an important place in the diagnosis and treatment of such lung diseases; however, the radiological changes in the lungs of people with COVID-19 pneumonia have not been fully characterized. The time between the onset of symptoms and the development of Acute Respiratory Distress Syndrome (ARDS) is as short as 9 days among the first patients with COVID19 pneumonia; that is why early diagnosis of the disease is essential for the treatment of these patients 6. Symptoms may emerge within 5–6 days after exposure to the agent; however, this period varies within the range of 2–14 days 3,5. While the majority of cases recover with mild symptoms; respiratory failure, acute respiratory distress, and death may be observed in severe cases 7. In particular, the disease is more severe in elderly male patients with medical comorbidities and smokers 8. The virus is mostly transmitted through close contact with infected people or the droplets generated by coughing, sneezing, and speaking 9. Furthermore, it is also stated that a person who comes into contact with a virus-infected surface/object can be infected in case they touch their mouth, nose, and eyes 9.

The management of COVID-19, for which there is no specific vaccine or antiviral treatment yet, consists of symptomatic treatment, supportive care, and isolation^10^. Measures should be taken to prevent the spread of the disease in fighting the outbreak. Therefore, various restrictions have been imposed in Turkey and these measures affect people's daily lives and cause economic and social problems. In this regard, the demographic data, disease symptoms, transmission route, treatment, and care results of the people with COVID-19 are of great importance for the healthcare workers in their efforts to control the outbreak and take necessary measures.

In this study, we aimed to examine the prevalence, clinical course, and outcomes of the patients with COVID-19 admitted to a state hospital in a city located in the southeastern part of Turkey.

## Methods

### Type, Place, and Time of the Study

This descriptive and cross-sectional study was carried out at a hospital, in Gaziantep, Turkey between March 16, 2020, and May 30, 2020 to retrospectively evaluate the results of COVID-19 surveillance.

### Sample of the Study

The population of the study consisted of the patients who applied to the surveillance unit of the state hospital and were placed in inpatient isolation. The data of the study was collected by retrospectively reviewing the files of 173 patients who were diagnosed with COVID-19 within the study period and were staying in the inpatient services, intensive care units, and a student dormitory used for the patient isolation. The patients who were referred to other hospitals (n=36) and whose file lacked the COVID-19 Case Information Form (n=5) were excluded from this study.

### Data Collection Tools

A retrospective review was carried out to collect the research data using the Covid-19 Case Information Form” in the patients' files. This form includes totally 15 questions and the following information: age, gender, whether having a chronic disease or not, unit of hospitalization, duration of hospitalization, time of the first COVID-19 symptom onset, whether there are patients with a similar clinical picture around, the history of being abroad, sample taking method and smoking status etc. In addition, the researchers followed the diagnostic test results from the patient electronic health records.

### Data Analysis

The data were analyzed using International Business Machines (IBM) Statistical Package for the Social Sciences (SPSS) Statistics for Windows, Version 22.0, Armonk, NY: IBM Corp. In the descriptive statistics, mean ± standard deviation was used in expressing the continuous numerical variables, and number (n) and percentage (%) in expressing the categorical variables. Pearson's Chi-Squared analysis was used in comparing the categorical variables and Bonferroni correction in finding which variable caused the difference. If the expected values were below 5 in (2x2) tables, Fisher's Exact Test was used to compare categorical data instead of Chi-Square Test. The patients' clinical outcomes were evaluated using Kaplan-Meier survival analysis. The statistical significance was set at p<0.05.

### Ethical Considerations of the Study

The study was started after receiving the required permissions from the following authorities: Faculty of Health Sciences, non-interventional Research Ethics Committee (Date: 07.05.2020, Decision No: 2020/028), The Provincial Directorate of Health, Gaziantep; the Chief Physician of Şehitkamil State Hospital, Gaziantep; and the Scientific Research Studies Unit, Directorate General for Health Services, Ministry of Health, Turkey.

## Results

The study was conducted with 173 patients diagnosed with COVID-19. Of these patients, 64.2% were male and 16.2% had a chronic disease. Their mean age was 34.76±25.75 years. The most common chronic disease was hypertension with 9.8%. Of the patients, 68.8% applied to the hospital due to the contact with a patient diagnosed with COVID-19 and 70.5% were being followed-up in an inpatient service. Considering that it would be easy to follow-up, the asymptomatic and stable COVID-19-positive patients were followed-up in the student dormitories prepared for pandemic patients. The patients who were diagnosed with COVID-19 had a good general condition and did not show any clinical symptoms (24.3%) were isolated and received treatment and care in a student dormitory affiliated to the hospital determined by the health directorates. Of the patients, 17.3% were smokers (n=30), and 71.1% had patients diagnosed with COVID-19 around them (n=123). As for the way of sample taking, combined nose and throat swabs were taken from 97.1% of the patients (n=168) and tracheal swabs from 2.3% of them since they were intubated (n=4). In the patients diagnoed with COVID-19, while the first samples were positive at a rate of 96.5% (n=167), this rate was found to be 22% (n=38) in the second test (Table 1).

**Table 1:** Patients' Descriptive information

Characteristics	n	%
**Mean Age:** 34.76±25.75		
**Gender**		
Male	111	64.2
Female	62	35.8
**Reason for Hospital Visit**		
Suspected	54	31.2
Contact with people having COVID-19	119	68.8
**Unit of Hospitalization**		
Service	122	70.5
Intensive care	9	5.2
Dormitory	42	24.3
**Chronic disease**		
Yes	28	16.2
No	145	83.6
Cardiovascular Disease*	8	4.6
Chronic Obstructive Pulmonary Disease*	7	4.0
Diabetes Mellitus*	10	5.8
Hypertension*	17	9.8
Cancer*	1	0.6
**Smoking status**	30	17.3
**Having patients diagnosed with COVID-19** **around**	123	71.1
**History of Travel Abroad**	1	0.6
**Way of Sample Taking**		
Combined Nose and Throat Swab	168	97.1
Throat Swab	1	0.6
Tracheal Swab	4	2.3
**Result of the 1^st^ Swab**		
Positive	167	96.5
Negative	6	3.5
**Result of the 2^nd^ Swab**		
Positive	38	22.0
Negative	131	75.7
Not taken	4	2.3

**Clinical status outcome**		
Ongoing Hospitalization	33	19.0
Recovered and Discharged	138	79.8
Exitus	2	1.2
**Duration of hospitalization:** Mean ±SD: 9.43±4.51 Median (IQR): 9 (8)		

* The patients marked more than one option.

When the patients' clinical symptoms at admission were analyzed, it was found that cough and fatigue (n=67) were the most common clinical symptoms with 38.7%, followed by headache with 23.7% (n=41), respiratory distress with 18.5% (n=32), and fever with 17.9% (n=31). Although 55.5% of the patients had a normal thorax CT, viral pneumonia was observed in 22.5% of them (Table 2). Of the patients, 65.3% were treated with Plaquenil tablets and used more than one medication during their hospital stay. (Table 3).

**Table 2:** Distribution of the patients' symptoms

COVID-19 Clinical Symptoms	n	%
How many days before the hospital visit did your clinical symptoms start? Mean± SD: 2.66± 2.767 (min:0 max:17)		
Fever*	31	17.9
Cough*	67	38.7
Distress*	32	18.5
Throat*	28	16.2
History*	12	6.9
Fatigue*	67	38.7
Headache*	41	23.7
Loss of Taste and Smell,*	11	6.4
Nausea/Vomiting*	7	4.0
Diarrhea*	9	5.2
Myalgia*	5	2.9
Abdominal Pain*	2	1.2
Thorax CT Result		
Normal	96	55.5
Icy Glass Appearance	22	12.7
Viral Pneumonia	39	22.5
Fibrotic Strips Extending to Pleura	16	9.3

* The patients marked more than one option.

**Table 3:** Distribution of medications used by the patients

Medications Used	n	%
**Hydroxychlorine sulfate** *		
Yes	113	65.3
No	60	34.7
**Enoxaparin Sodium** *		
Yes	110	63.6
No	63	36.4
**Oseltamivire** *		
Yes	37	21.4
No	136	78.6
**Ceftriaxone** *		
Yes	23	13.3
No	150	86.7
**Moxifloxacin** *		
Yes	33	19.1
No	140	80.9

* Patients used more than one medication.

**Graph 1:**
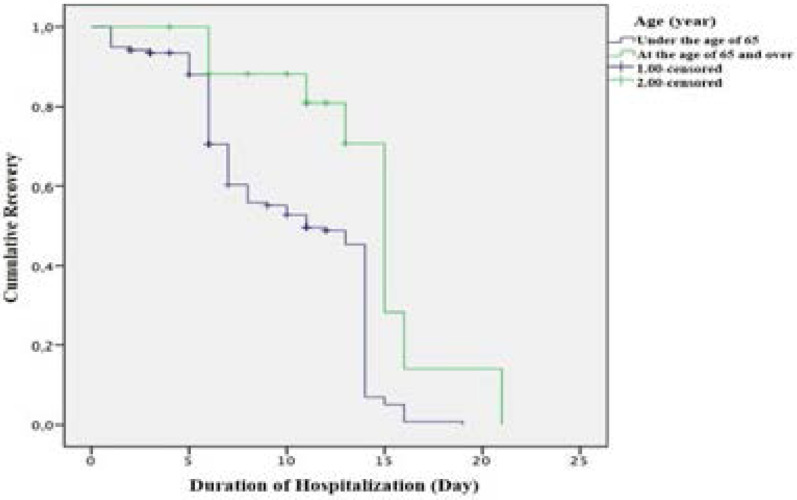
Comparison of the patients' recovery and discharge rates by age groups (Kaplan-Meier Curve)

As for the patients' clinical outcome, it was found that 79.8% of the patients were discharged after recovery, 1.2% of them (2 patients) died, and the others' treatments were ongoing (Table 1). The survival analysis revealed that the patients' cumulative survival rate in the 1st month was found to be 97.6%±0.9%. When the patients' recovery and discharge rates were compared by age groups, it was found that the mean discharge time for the patients under the age of 65 was 10.17±0.369 days (at confidence interval of 95%; lower and upper limits were 9.448 and 10.897, respectively), and the mean discharge time for the patients at the age 65 and over was 14.436±1.223 (at confidence interval of 95%; lower and upper limits were 12.039 and 16.832, respectively); so, the patients at the age 65 and over were discharged later (x2=9.894, p=0.002) (Graph 1).

When some surveillance results were compared by the age groups, it was found that the patients under the age of 65 were admitted to the hospital mostly due to contact with other people with the disease, and the patients at the age of 65 and over were treated mostly in the intensive care unit (p=0.006 and p<0.001, respectively). When the patients' symptoms due to COVID-19 were evaluated by the age groups, it was found that the patients at the age of 65 and over had statistically significantly more dyspnea, nausea/vomiting, and cardiovascular symptoms (p=0.007, p=0.026, and p=0.038, respectively). It was observed that the patients at the age of 65 and over had more chronic diseases; on the other hand, more patients under the age of 65 had people with similar symptoms around them and they had statistically significantly more positive symptoms in the thoracic CT (p<0.001, p=0.004, and p<0.001, respectively) (Table 4). When the patients' some surveillance results were compared by gender; it was found that the women were isolated mostly in pandemic services and the men mostly in the dormitories (p=0.033). It was found that while there was no statistically significant difference between the genders in terms of disease symptoms (p>0.05), more women diagnosed with COVID-19 had chronic diseases than the men (p=0.010) (Table 4).

**Table 4:** Comparison of some surveillance data by age groups and gender (n=173)

	Under the age of 65 n(%)	65 years and over n(%)	Test (*x*^2^) p	Male n(%)	Female n(%)	Test (*x*^2^) p
**Reason for Hospital Visit**						
Suspected	43 (27.7)	11 (61.1)	0.006*†	31(27.9)	23(37.1)	1.558 0.212
Contact with people having COVID-19	112 (72.3)	7 (38.9)		80(72.1)	39(62.9)	
**Unit of Hospitalization**						
Service ^a^	108 (69.7)	14 (77.8)	16.214 <0.001** (a-b,c)	72(64.9)	50(80.6)	6.844 0.033* (a-c)
Intensive care ^b^	5 (3.2)	4 (22.2)		5(4.5)	4(6.5)	
Student Dormitory ^c^	42 (27.1)	0 (0)		34(30.6)	8(12.9)	
**Fever**						
Yes	26(16.8)	5 (27.8)	0.325†	19(17.1)	12(19.4)	0.135 0.713
No	129(83.2)	13 (72.2)		92(82.9)	50(80.6)	
**Cough**						
Yes	57(36.8)	10(55.6)	0.133†	43(38.7)	24(38.7)	0.001 0.997
No	98(63.2)	8(44.4)		68(61.3)	38(61.3)	
**Dyspnea**						
Yes	24(15.5)	8(44.4)	0.007*†	20(18.0)	12(19.4)	0.047 0.828
No	131(84.5)	10(55.6)		91(82.0)	50(80.6)	
**Sore throat**						
Yes	26(16.8)	2(11.1)	0.741†	17 (15.3)	11(17.7)	0.173 0.678
No	129(83.2)	16(88.9)		94 (84.7)	51(82.3)	
**Headache**						
Yes	36(23.2)	5(27.8)	0.770†	27(24.3)	14(22.6)	0.067 0.796
No	119(76.8)	13(72.2)		84(75.7)	48(77.4)	
**Nausea/Vomiting**						
Yes	4(2.6)	3(16.7)	0.026*†	3(2.7)	4(6.5)	0.251†
No	151(97.4)	15(83.3)		108(97.3)	58(93.5)	
**Fatigue**						
Yes	57(36.8)	10(55.6)	2.397 0.122	43(38.7)	24(38.7)	0.001
No	98(63.2)	8(44.4)		68(61.3)	38(61.3)	0.997
**Diarrhea**						
Yes	7(4.5)	2(11.1)	0.237†	6(5.4)	3(4.8)	0.871†
No	148(95.5)	16(88.9)		105(94.6)	59(95.2)	
**Abdominal Pain**						
Yes	2(1.3)	0	1.00†	1(0.9)	1(1.6)	1.00†
No	153(98.7)	18(100)		110(99.1)	98.4)	
**Myalgia**						
Yes	4(2.6)	1(5.6)	0.427†	2(1.8)	3(4.8)	0.351†
No	151(97.4)	17(94.4)		109(98.2)	59(95.2)	
**Cardiovascular Symptoms**						
Yes	5(3.2)	3(16.7)	0.038*†	4(3.6)	4(6.5)	0.459†
No	150(96.8)	15(83.3)		107(96.4)	58(93.5)	
**Loss of taste and smell**						
Yes	9(5.8)	2(11.1)	0.320†	6(5.4)	58.1)	0.472 0.492
No	146(94.2)	16(88.9)		105(94.6)	57(91.9)	
**Chronic disease**						
Yes	18(11.6)	10(55.6)	22.956 <0.001**	12(10.8)	16(25.8)	6.594 0.010*
No	137(88.4)	8(44.4)		99(89.2)	46(74.2)	
**Are there any person(s) with similar disease setting around you?**						
Yes	116(74.8)	7(38.9)	0.004*†	81(73.0)	42(67.7)	1.240 0.538
No	38(24.5)	11(61.1)		29(26.1)	20(32.3)	
**Is there any positive sign in Thorax CT?**						
Yes	76(49.0)	1(5.6)	<0.001**†	54(48.6)	23(37.1)	2.149 0.143
No	79(51.0)	17(94.4)		57(51.4)	39(62.9)	
**Clinical status outcome**						
Discharged	129(83.2)	9(50.0)	12.374 0.002*	91(82.0)	47(75.8)	2.634 0.268
Ongoing Treatment	25(16.1)	8(44.4)		18(16.2)	15(24.2)	
Exitus	1(0.6)	1(5.6)		2(1.8)	0	

* p<0.05

** p<0.001 was accepted as statistically significant.

x^2^=Pearson's Chi-squared Test

† Fisher's Exact Test

## Discussion

In this study, the clinical course and outcome of 173 patients diagnosed with COVID-19 were examined in a state hospital located in the southeastern part of Turkey. It was determined that the number of male patients was higher than that of female patients. Likewise, when the previous studies were reviewed, it was seen that the data results of a meta-analysis including the results of 15 observational sudies examining the distribution of COVID-19 by gender across the world have showed that the rate of the males infected by COVID-19 is higher than the females 11. In the study carried out by Mengmeng Zhao et al., it was determined that 53.4% of 1000 patients diagnosed with COVID-19 were female 12. In a study carried out by Jian Wu et al., it was revealed that 51.25% of 80 patients diagnosed with COVID-19 were female 13. This varies depending on the culture and may stem from the fact that the males in the region where the hospital is located are the ones who earn a livelihood for their families, so they spend more time outside home and have contact with more people.

It is a proven finding that there is a relationship between COVID-19 and age in terms of clinical outcomes. The studies carried out across the world have revealed that as the age increases, the course of COVID-19 gets more severe 12. In the present study, the mean age of the sample was found to be 34.76 years. At the beginning of the outbreak, it was reported that the mean age for 214 patients diagnosed with COVID-19 at Wuhan Huazhong University Hospital was 52.7 14. Guan et al. reported the mean age as 47 and stated that as the age increased, the rate of having a concomitant chronic disease also increased in the patient diagnosed with COVID-19 15. In the study by Zhao et al., the mean age for 1000 patients diagnosed with COVID-19 was found to be 61, and it was observed that having concomitant chronic diseases became more risker as the age increased 12. Age has been the most prominent factor in the COVID-19 process. However, it is worthy of note that the rate of young population in each country is different in the studies examining the age factor.

One of the important findings was that the patients with COVID-19 had chronic diseases, especially hypertension. In other reports revealed across the world and a study involving 269,070 patients at the age of 65 and over in the UK and examining chronic diseases within the context of COVID-19, it has been asserted that the most common chronic disease in the patients with COVID-19 is hypertension, followed by coronary heart disease and diabetes. However, it was emphasized that there was no causal relationship between COVID-19 and hypertension or other chronic diseases 16. Because it is noted that high prevalence of these chronic diseases in elderly people is considered normal 16. The relationship between COVID-19 and chronic diseases has not been fully clarified and there is a need for further study in this regard.

One of the important findings obtained in the present study is the clinical symptoms in patients diagnosed with COVID-19. The most common symptom observed in the sample was cough and fatigue, followed by headache, respiratory distress, and fever. Likewise, according to the data of CDC and other international studies, it has been reported that patients show symptoms such as fever, cough, and shortness of breath 17–22. According to the data provided by Statista, German online official statistics portal, (July-2020); the most common symptom in Italy, most severely affected country by COVID-19 in Europe, is fever, followed by shortness of breath and cough 17. In the statistical analyses on the patients with COVID-19 in China, it has been observed that fever is the most common symptom with, followed by dry cough and fatigue^18^. Nurses should focus on symptom management in patients diagnosed with COVID-19. Symptom management; which starts with identifying the patients' symptoms, following-up the vital signs, and providing bed rest; can be considered as the first step of nursing care for patients diagnosed with COVID-19 20. In order to provide a holistic nursing care and to take a systematic approach, it is essential for nurses and all healthcare professionals to know which symptom can be at which severity and their physiopathology 21. Moreover, holistically evaluating not only the common symptoms but also new or previously undiscovered or rare symptoms will ensure a smooth treatment and care for the patients.

It was found that the most common reason for contracting the disease is “having contact with other patients previously diagnosed with COVID-19”. According to several reports mentioned in another study on this subject, it has been confirmed that COVID-19 transmits from person to person, and it is asserted that the disease spread by the droplets generated by coughing, sneezing, and speaking, especially in close contact [within 7 feet (2 meters)] 22. Therefore, nurses and patients diagnosed with COVID-19 should focus on preventing the spread of infection to other staff and patients, and strict rules should be imposed in terms of the measures to be taken 23.

It was found that while the patients at the age of 65 and over were treated in intensive care units and pandemic services, the young patients were mostly isolated in student dormitories and treated in the services. The reason for this is that elderly patients had more severe symptoms due to their chronic diseases and weak immune systems, and therefore they were more dependent on others in their daily living activities; so, they were followed-up in the services and intensive care units 5,8,21. Moreover, although not surprising, this was supported by the fining that the patients at the age of 65 and over in this study had more chronic diseases, respiratory distress, and cardiovascular and gastrointestinal symptoms. On the other hand, the fact that more male patients were followed-up in dormitories suggests that the disease progresses asymptomatically or with mild symptoms mostly in the male patients. Furthermore, the female patients in this study had statistically significantly more chronic diseases than the males, and this can be the reason for their being followed-up in the intensive care unit and service.

The thoracic CT diagnostic test revealed a striking finding: the patients at the age of 65 and over and the female patients are often diagnosed with COVID-19 without having any symptoms. In this study, the thorax CT results were available for all cases which were examined retrospectively. Computed tomography has been reported to be the most preferred diagnostic test in the diagnosis of COVID-19 since the symptoms such as fever, cough, fatigue, headache, and shortness of breath are not specific to the disease which can rapidly progress to a severe pneumonia and the real-time reverse transcription polymerase chain reaction (RT-PCR) test may give false negative results, although it is the gold standard in the diagnosis of COVID-19 as a powerful diagnostic test 24–26. However, it has also been emphasized that CT should be used as a problem-solving method in the patients with negative RT-PCR test result rather than a screening method, especially because it contains ionizing radiation 26,27. In the light of this information, the physicians, nurses, and all other healthcare professionals should take into consideration that the thoracic CT diagnostic test applied to the patients without any viral pneumonia signs will not yield the desired results. Furthermore, in this study, the fact that the COVID-19 symptoms were less observed in the thorax CT results of the male patients due to isolation in student dormitories may be put forward as an evidence that the male patients experience milder symptoms; and in the patients at the age of 65 and over, the physicians might have applied this diagnostic test not to overlook the diagnosis of COVID-19, not to confuse the symptoms with those of other diseases, and to be sure of the diagnosis.

As for the patients' clinical outcomes, it was found that most of the patients were discharged after recovery, 1.2% of them (2 patients) died, and the others' treatments were ongoing. The survival analysis revealed that the patients' cumulative survival rate in the 1st month was found to be 97.6%±0.9%. As for the comparison of the patients' recovery and discharge rates by age groups, it was found that the mean discharge time for the patients under the age of 65 was about 10 days and the mean discharge time for the patients at the age 65 and over was about 15 days; so, the patients at the age 65 and over were discharged later (Graph 1). Zheng et al. (2020) reported that 12.7% of the patients died and those who died had been hospitalized for 11 days on average. The same study also reported that the mortality rate was higher in the male and elderly patients, and the mean treatment duration in the surviving patients was 22 days in those under the age of 40, 34 days in those at the age of 80 and over 28. It is thought that the reason why the hospital stay is prolonged with age in the patients diagnosed with COVID-19 is because their immune system is weak and they have more chronic diseases.

## Limitations

This study had some limitations in that it was a single-centered retrospective study just based on the information obtained from patient records and only covered a 3-month-period for the COVID-19 positive cases.

## Conclusions

In this study, it was found that the majority of the patients were male; the most common symptoms were cough, fatigue, headache, respiratory distress, and fever. It was determined that two patients (1.2%) died, the patients under the age of 65 had a shorter hospitalization period and recovered and were discharged in a shorter time. It was found that there were differences between the age groups in terms of transmission route, the clinic where they were being followed-up, some symptoms, thorax CT result, and clinical status outcome. Moreover, the male patients were mostly followed-up in student dormitories and the female patients had more chronic diseases. In the light of all these findings, it is considered that clarifying the COVID-19 symptoms and the affecting factors will guide all healthcare personnel in the treatment and care to be designed for this disease, which took hold across the world. On the other hand, it is recommended that multi-center, prospective, generalizable, experimental or observational studies with larger samples be carried out and the patients be followed-up for longer periods in a way to eliminate the limitations of this study.
